# Underlying Causes and Therapeutic Targeting of the Inflammatory Tumor Microenvironment

**DOI:** 10.3389/fcell.2018.00056

**Published:** 2018-06-12

**Authors:** Elizabeth A. Comen, Robert L. Bowman, Maria Kleppe

**Affiliations:** ^1^Breast Cancer Service, Department of Medicine, Memorial Sloan Kettering Cancer Center, New York, NY, United States; ^2^Center for Hematopoietic Malignancies, Memorial Sloan Kettering Cancer Center, New York, NY, United States; ^3^Human Oncology and Pathogenesis Program, Memorial Sloan Kettering Cancer Center, New York, NY, United States

**Keywords:** chronic inflammation, clonal hematopoiesis, stroma, microenvironment, tumor suppressors, oncogenes, anti-inflammatory drugs

## Abstract

Historically, the link between chronic inflammation and cancer has long been speculated. Only more recently, pre-clinical and epidemiologic data as well as clinical evidence all point to the role of the tumor microenvironment as inextricably connected to the neoplastic process. The tumor microenvironment (TME), a complex mix of vasculature, inflammatory cells, and stromal cells is the essential “soil” helping to modulate tumor potential. Increasingly, evidence suggests that chronic inflammation modifies the tumor microenvironment, via a host of mechanisms, including the production of cytokines, pro-inflammatory mediators, angiogenesis, and tissue remodeling. Inflammation can be triggered by a variety of different pressures, such as carcinogen exposure, immune dysfunction, dietary habits, and obesity, as well as genetic alterations leading to oncogene activation or loss of tumor suppressors. In this review, we examine the concept of the tumor microenvironment as related to both extrinsic and intrinsic stimuli that promote chronic inflammation and in turn tumorigenesis. Understanding the common pathways inherent in an inflammatory response and the tumor microenvironment may shed light on new therapies for both primary and metastatic disease. The concept of personalized medicine has pushed the field of oncology to drill down on the genetic changes of a cancer, in the hopes of identifying individually targeted agents. Given the complexities of the tumor microenvironment, it is clear that effective oncologic therapies will necessitate targeting not only the cancer cells, but their dynamic relationship to the tumor microenvironment as well.

## Introduction

Chronic inflammation is a hallmark of cancer and many factors can trigger an inflammatory response in the microenvironment, including infectious pathogens, imbalanced immune regulation, carcinogen exposure, dietary habits and obesity, and genetic alterations leading to oncogene activation or loss of tumor suppressors (Elinav et al., 2013). A growing understanding of the relationship between chronic inflammation and the tumor microenvironment (TME) has dramatically altered our understanding of cancer. Evidence suggests that chronic inflammation creates a pro-tumorigenic environment via the production of pro-inflammatory mediators, angiogenesis, and tissue remodeling (Coussens and Werb, 2002). Some of the most critical external factors that can promote chronic inflammation and increase cancer risk include tobacco, obesity, a sedentary lifestyle, and select infectious agents. Evolving insight into the mechanisms by which chronic inflammation supports a pro-tumorigenic environment has led to new (immuno)-therapies for cancer as well as lifestyle recommendations which may decrease cancer incidence.

**Figure 1:**
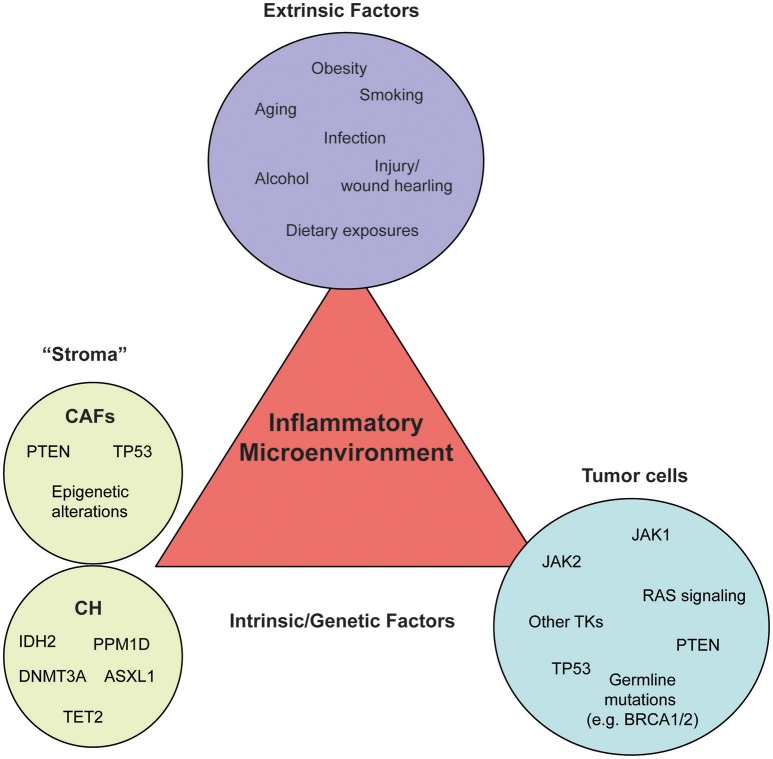
External and intrinsic factors fueling an inflammatory microenvironment. Inflammation can be triggered by a variety of different pressures, such as carcinogen exposure, immune dysfunction, dietary habits, and obesity (extrinsic factors, purple circle), as well as genetic alterations (intrinsic factors) leading to oncogene activation or loss of tumor suppressors in the microenvironment (stroma, green circle) or the tumor cells themselves (tumor, blue circle). Each circle contains a number of examples which belong to the respective category. Most genetic and epigenetic alterations of the stroma have been identified in cancer-associated fibroblasts (CAFs) and white blood cells of healthy individuals or solid cancer patients without signs of hematological malignancies (CH).

In this review, we discuss the current knowledge of how epithelium cancer-initiating events cross talk to inflammatory cells during cancer initiation and progression. Specifically, we review the concept of the TME and both the extrinsic and intrinsic mechanisms that tie an inflammatory response to pro-tumorigenic events (Figure 1).

## Cellular constituents of an inflammatory microenvironment

While the exact composition of the TME will differ from tissue to tissue, as well as between various tumors of the same tissue, key cellular players are recurrently found. In addition to malignant tumor cells, the TME is composed of a number of different cell types including fibroblasts, endothelial cells, pericytes, and various cells associated with the immune system (Quail and Joyce, 2013). In addition to B cells, natural killer (NK) cells and T lymphocytes, the myeloid cells within the TME include tumor-associated macrophages (TAMs), dendritic cells, neutrophils, and monocytes, the latter two of which have often been misclassified as myeloid-derived suppressor cells (MDSCs) on the basis of immunophenotypic markers. In mice, these myeloid cells are identified by immunophenotype (Cd11b^+^, Gr.1^+^) with Ly6C and Ly6G to further differentiate a monocytic-MDSC from a granulocytic-MDSC respectively (reviewed here Talmadge and Gabrilovich, 2013). Human MDSCs are defined through a combination of CD11B, CD14, CD15, and CD66 (Bronte et al., 2016). While studies have identified bona fide T cell suppressor function within this compartment, the label of MDSC is often applied even without functional demonstration leading to murky interpretations of their role within the TME. Understanding the molecular functions of these cells within the tumor may provide avenues for disrupting pro-tumorigenic signaling and tailored TME-targeted therapy. Furthermore, identifying markers capable of distinguishing functional MDSC from the immunophenotypically similar monocyte and neutrophil counterparts will be critical to elucidating their role in the TME (Bronte et al., 2016). In this section, we will review the functions of three of the most well studied constituents of the TME, TAMs, cancer-associated fibroblasts (CAFs) and endothelial cells comprising the tumor vasculature.

### Tumor-associated macrophages

TAMs have emerged as one of the most well studied components of the TME. These cells have been shown to interact with nearly every feature of tumor progression including mediating tumor cell proliferation, migration, invasion, angiogenesis, metastasis, and chemotherapeutic resistance (Noy and Pollard, 2014). The multifaceted role of TAMs in the TME is a direct reflection of the diverse responsibilities of macrophages in normal development, tissue homeostasis, and tissue repair (Okabe and Medzhitov, 2016). Indeed many of the developmental processes mediated by macrophages, including extracellular matrix remodeling, phagocytosis of apoptotic debris and angiogenesis, are critical hallmarks of the TME. As such, in some instances the inflammatory state of the tumor resembles an unresolved wound healing response (Dvorak, 1986; Schäfer and Werner, 2008). Perhaps the most well described function of TAMs involves their role in tumor cell migration and invasion. TAMs have been shown to engage in a CSF1-EGF paracrine signaling loop capable of leading tumor cells to the invasive edge of a tumor (Wyckoff et al., 2004). Additionally, TAMs have been shown to engage in a multicellular interaction with endothelial cells through the release of VEGFA to facilitate the intravasation of tumor cells from the primary site into circulation (Harney et al., 2015). In addition to the production of these growth factors, TAMs are the major source of matrix metalloproteinases and cathepsin proteases (Olson and Joyce, 2015; Varol and Sagi, 2017). In order to execute the diverse functions described above, macrophages employ immense transcriptional plasticity that falls along a continuum of activation (Xue et al., 2014; Glass and Natoli, 2016). On one extreme end of the spectrum lays classically activated macrophages (often termed M1-macrophages mirroring that of Th1 immunity). This activation state is canonically associated with IFNγ stimulation resulting in a STAT1 transcriptional program. Functionally, these cells are capable of perpetuating type I inflammatory responses through the secretion of chemokines such as CCL3, CCL4, and CCL5, as well as the production of nitric oxide and TNFα. Furthermore, IFNγ stimulation can boost antigen presentation capacity through the upregulation of *Ciita* a master transcriptional regulator of MHC II molecules. On the other extreme, alternatively activated macrophages (or M2 macrophages) are associated with an anti-inflammatory state. Here, the critical molecular regulators of alternative activation are the Th2 cytokines IL-4, IL-13, IL-6, and IL-10. Together these cytokines lead to the activation of STAT6 (IL-4 and IL-13) as well as STAT3 (IL-6 and IL-10). These programs, as well as other transcriptional regulators, drive the upregulation of Arginase 1 leading to decreased nitric oxide signaling, and increased expression of the wound healing associated chemokines CCL17 and CCL22. Functionally, this activation state is associated with inflammation resolution and extracellular matrix remodeling. In addition to the cytokines described above, TAM activation can be influenced by hypoxia and local metabolite concentrations such as lactate (Casazza et al., 2013; Colegio et al., 2014; Carmona-Fontaine et al., 2017). Despite the widespread description of TAMs as either M1 or M2, this dichotomy is clearly an oversimplification of the diverse states in which these cells are capable of existing (Murray et al., 2014). Elucidating the functional capacities of TAMs within the TME and the mechanism that regulate these processes will provide a clearer picture of these cells. Another more recently appreciated factor influencing TAM activation involves the origin of the cells. While TAMs were long thought to derive from circulating monocytes (Qian et al., 2011; Franklin et al., 2014), recent work suggests that TAMs are also derived from local tissue-resident macrophages (Bowman et al., 2016; Zhu et al., 2017). These are important to consider in the setting of therapeutic strategies aimed at reducing TAM accumulation through recruitment blockade, either through CCR2 or CXCR4 inhibition (Kioi et al., 2010; Qian et al., 2011). Further, in most cases, tissue-resident macrophages possess distinct developmental origins from their monocyte-derived counterparts, as they seed the tissue during embryogenesis developing via an erythro-myeloid precursor as opposed to a hematopoietic stem cell (Gomez Perdiguero et al., 2015). This distinct ontogeny appears to imprint a sort of epigenetic memory on the subsequent TAM, eliciting distinct gene expression profiles within the TME (Bowman et al., 2016; Zhu et al., 2017). While clodronate liposome-based depletion strategies have been used to preferentially deplete tissue resident macrophages in the pancreas (Zhu et al., 2017), more selective genetic ablation strategies will be of interest to translate the differences seen in gene expression studies into functional capacities. Translation of these studies from the mouse to human disease will require identification of markers capable of distinguishing the ontogenetically defined TAM populations. One such marker, CD49D, has been found to be absent on brain-resident microglia and present on recruited bone marrow-derived macrophages in multiple brain malignancies (Bowman et al., 2016). Markers such as CD49D will likely be found in many distinct tissues, and may serve as biomarkers for future TME-targeted therapy.

### Cancer-associated fibroblasts (CAFs)

In addition to the immune components of the TME described above, CAFs are an abundant, heterogeneous pool of cells that play multifactorial roles in cancer progression. CAFs are sometimes referred to as mesenchymal stem cells or tumor-associated fibroblasts (Paunescu et al., 2011). Regardless of the nomenclature, these cells are non-hematopoietic, non-epithelial cells resident to a tissue. These cells can be identified microscopically based on the spindle-like shape and large singular presence within the stroma of a tissue. During tissue homeostasis, these cells are responsible for deposition of type I collagen, laminin, perlecan, nidogen, and fibronectin, but are generally considered quiescent with limited migration and proliferation (Kalluri, 2016). Much like macrophage activation paradigms described above, fibroblasts undergo a similar activation process upon stimulation with factors such as TGFβ, PDGF, and FGF2 (Elenbaas and Weinberg, 2001). Like the TAMs described above, CAFs are distinct from their non tumor-associated counter parts and possess a unique activation state. Upon activation these cells change morphologically, increasing in size and with additional spindle-like processes. Functionally these cells possess increased capacity for migration, collagen crosslinking and secretion of cytokines and chemokines such as VEGFA, TGFβ, HGF, FGF, EGF, CXCL10, CCL5, IL-6, TNFα, and IFNγ (Kalluri, 2016). Through these phenotypic alterations, activated fibroblasts can orchestrate a wound healing response concomitant with extracellular matrix repair, recruitment of immune cells to eliminate pathogens, and regrowth of damaged epithelial tissue (Öhlund et al., 2014). Critically, fibroblast activation is a reversible process and as such wound healing responses are able to resolve and quiescence can be restored. If however, this process is not resolved tissue fibrosis can occur. In cancer, this fibrotic phenotype is widespread with even premalignant lesions are often associated with fibrosis or desmoplastic reactions (Rønnov-Jessen et al., 1996), however the causality of desmoplasia and malignant transformation remain an open discussion in human disease. In developed tumors, pancreatic adenocarcinoma (PDAC) presents an extreme example of unrestrained desmoplasia, driven in part through sonic hedgehog signaling in the stroma (Tian et al., 2009). The fibrotic stroma of PDAC presents a challenge for effective delivery of chemotherapy into tumors, and as such reducing the stromal component of the TME presents an interesting chemo sensitizing therapeutic option (Olive et al., 2009). A more full understanding of how CAFs are activated, and the results of inhibiting these activation states are necessary. While most studies have been completed *in vitro*, one study utilized genetic mouse models of squamous cell carcinoma. CAFs isolated from these early neosplasms possess a pro-inflammatory gene expression signature driven by NF-κB (Erez et al., 2010). Interestingly, the authors demonstrated that normal dermal fibroblasts could be “educated” to resemble CAFs through co-culture with carcinoma cells. This activation state has since been shown to be regulated through promoter hypermethylation (Zeisberg and Zeisberg, 2013; Li et al., 2015; Xiao et al., 2016). Upon activation CAFs interface with the tumor through many of the same mechanisms as an activated fibroblast during wound healing, yet many differences remain. *In vitro* proteomic studies identified an altered secretory phenotype in CAFs compared to non-malignant activated fibroblasts (De Boeck et al., 2013). In this study, CAFs were found to secrete higher levels of tenascin and the CXCR4 ligand SDF-1, both of which have been shown to be important in different stages of the metastatic cascade (Oskarsson et al., 2011; Vanharanta et al., 2013). Additionally, while one of the primary functions of fibroblasts in wound healing is to recruit immune cells, CAFs have been shown to negatively regulate immune responses in a TNFα and IFNγ dependent manner (Kraman et al., 2010). As such, targeting CAF-derived cytokines has been shown to enhance CSF1R targeted therapy (Kumar et al., 2017) as well as immune checkpoint blockade (Feig et al., 2013). Clinical efficacy of these combinations remains to be determined.

### Angiogenesis and tumor vasculature

The formation of new blood vessels, termed angiogenesis, is one of the hallmarks of the tumor microenvironment (Hanahan and Weinberg, 2011). The newly formed vascular network serves as a means to deliver nutrients, cytokines, and oxygen into the tumor. Thus engaging in angiogenesis is a major step in disease development, with earlier stage, smaller tumors possessing fewer vessels than later, more aggressive tumors, which can be highly vascularized (Bergers and Benjamin, 2003). This is, of course, a broad generalization as the vascular content also varies by tissue. For example, while glioblastoma multiforme is one of the most vascularized tumor types (Das and Marsden, 2013), pancreatic ductal adenocarcinoma has low microvascular density and is instead entrenched with dense desmoplastic stroma (Longo et al., 2016). Unlike its' normal tissue counterpart, tumor vasculature often possesses aberrant morphology associated with increased branching and an overall disrupted network of endothelial cells. These abnormal structural findings are often caused by poor pericyte coverage and disruption of a supportive basement membrane (De Palma et al., 2017). Such a distorted network can lead to poor diffusion of oxygen and other small molecules in the tumor resulting in spatial heterogeneity.

While a critical process in malignant development, angiogenesis is not unique to tumors, but rather reminiscent of a wound healing response initiated by inflammation. Indeed, recruited monocytes, eosinophils, and neutrophils are capable of secreting pro-angiogenic factors (De Palma et al., 2017). One of the most potent inducers of angiogenesis is hypoxia, which activates hypoxia inducible factor 1 (HIF1) in both tumor cells and surrounding stromal cells leading to the production of vascular endothelial growth factor (VEGF), a potent mediator of new vessel growth (Krock et al., 2011). In addition, TAMs and CAFs are also capable of stimulating angiogenesis through the secretion of VEGFA as well as the lymphangiogenic factor VEGFC and VEGFD (Quail and Joyce, 2013). Given its critical role in angiogenesis, VEGF-targeted agents have emerged as a major class of therapeutics with broad applicability in cancer (Ferrara and Adamis, 2016). Despite success in some tumor types (renal cell carcinoma), understanding which patients are most likely to benefit from anti-angiogenic therapy remains a challenge. Angiogenic signaling is further complicated in the context of inflammation where the paralogous factors angiopoietin-1 and angiopoietin-2 play a role in dampening and amplifying sensitivity to the inflammatory factor TNFα during wound healing (Fiedler et al., 2006). Two recent studies demonstrated that dual targeting of angiopoietin-2 and VEGF resulted in vascular normalization and extended survival in murine models of glioblastoma (Kloepper et al., 2016; Peterson et al., 2016). This combination was also found to potentiate immunotherapy via PD-1 blockage (Schmittnaegel et al., 2017). Understanding the interplay between these molecules and the tumor's dependency will be critical for maximizing future anti-angiogenic therapeutic approaches. While the vasculature plays a clear role in delivering nutrients to a tumor, several reports have provided evidence for an additional role in promoting cancer stem cells. In colorectal cancer (CRC) endothelial cells have been shown to support cancer stem cells through soluble Jagged-1 mediated activation of the Notch signaling (Lu et al., 2013). Similar results were found for Jagged-1, as well as DLL4, in glioblastoma models (Zhu et al., 2011). These studies suggest that targeting of endothelial cells may provide an avenue for modulating cancer stem cells; however, previous reports have demonstrated that anti-angiogenic therapies can actually lead to an increase in cancer stem cells due to increased hypoxia (Conley et al., 2012). Given the multifaceted role of the tumor vasculature, careful preclinical studies will be necessary to understand the consequences of endothelial-targeted therapy.

In addition to supporting primary tumor growth, endothelial cells are also involved in the metastatic cascade from the earliest step of intravasation to vascular cooption at the metastatic site. Histological interrogation identified a tri-cellular signaling hub known as TMEM structure, composed of a tumor cell, a Tie2^high^ TAM and an endothelial cell (Robinson and Jones, 2009). Further intravital imaging studies revealed that at this site local VEGF signaling leads to increased vascular permeability followed by a release of tumor cells into the blood stream (Harney et al., 2015). Later in the metastatic cascade, during extravasation, endothelial cells can serve as a barrier between the circulating tumor cells and host tissue. In brain metastasis for instance, cleavage of the endothelial adhesion molecule, JAM-B, is necessary for tumor cells to efficiently extravasate (Sevenich et al., 2014). Upon extravasation, metastatic cells once again rely upon close association with endothelial cells to support their survival in a process known as vascular cooption. These studies collectively highlight that endothelial cells play critical roles in both tumor development through increased vascularization, but are also intricately involved in many steps of disease progression.

## Causes of an inflammatory microenvironment

### External factors and exposures cause acute and chronic inflammation

Evidence indicates that chronic inflammation not only increases the risk of a multitude of cancers, including colon, liver, pancreatic, lung, bladder, gastric, and breast, but may also increase the risk of tumor progression and metastasis (Iyengar et al., 2015). Moreover, it is well known that a variety of external factors and exposures can cause both acute and chronic inflammation. Because many of these factors/exposures, such as diet, are modifiable, there is a growing scientific and public health interest in understanding the relationship between extrinsic pressures that promote chronic inflammation and the TME.

### Tobacco

Historically, tobacco has been the most notorious carcinogen. At present, tobacco exposure is the leading preventable cause of death. Roughly 85% of all lung cancers are secondary to smoking with additional cancers attributable to secondary smoke (Warren and Cummings, 2013). In developed countries, tobacco is associated with roughly 30% of all malignancies (McGuire, 2016). Tobacco use is associated with numerous other cancers as well, including but not limited to, head and neck, pancreatic, gastric, esophageal, acute myeloid leukemia (AML), bladder and renal cell cancers (United States Public Health Service, and Office of the Surgeon General, 2010). For example, actively smoking triples the risk of bladder cancer (Freedman et al., 2011; McGuire, 2016). Tobacco increases cancer risk not only by causing direct genetic changes to the epithelium (particularly the lung), but also through altering epigenetic events, eliciting epithelial to mesenchymal cell transition, and inducing a chronic inflammatory and hypoxic microenvironment. Ongoing inflammation promotes apoptotic arrest, angiogenesis and in turn cell proliferation (Milara and Cortijo, 2012). Tobacco inherently contains a multitude of direct carcinogens, including polycyclic aromatic hydrocarbons, *N*-nitrosamines, aromatic amines, aldehydes, volatile organic hydrocarbons, and metals among others (Pfeifer et al., 2002). The activation of these carcinogens in the host can result in the formation of DNA adducts, a form of DNA damage and source of mutagenesis. In addition to directly affecting DNA function of the cell, tobacco can modify the immune function of the host (Sopori, 2002). For example, tobacco can increase the abundance of alveolar macrophages in the lung, which may increase oxidative stress and oxygen radicals, thereby promoting tumor growth. In a study of 1,819 individuals, systemic levels of 78 makers of inflammation and immunity were measured. The study included 548 never smokers, 857 former smokers, and 414 current smokers. Significant differences in several immune markers were noted between active and never smokers. These include but are not limited to differences in CCL17/TARC, CCL11/EOTAXIN, IL-15, IL-1B, IL-1Rα, CRP, SVEGFR3, IL-16, sIL-6R, and SCF. Interestingly, the authors note that many of these makers are critical to immune function as well as cell growth. Overall, they report that on average smokers appear to have an immune profile consistent with an overall immunosuppressive function of tobacco (Shiels et al., 2014). Indeed multiple studies have demonstrated that tobacco has immunosuppressive functions (Cui and Li, 2010). One mechanism by which nicotine may be immunosuppressive is by impairing macrophage and neutrophil function (Milara and Cortijo, 2012). In tobacco smokers with chronic obstructive airway disease (COPD) the lung epithelium undergoes repeated injury and repair thereby inducing transformation of the normal epithelium to a more malignant phenotype. Tobacco smokers who continue to smoke also have poorer outcomes from a multitude of cancers, perhaps as a result of suppression of NK cell activation (Lu et al., 2007). Despite the significant link between tobacco and lung cancer, some non-smokers still develop lung cancer. Toward this end, understanding the mechanisms by which smokers as well as non-smokers may share common markers related to chronic airway inflammation may shed important light into molecular targets for the diagnosis and treatment of lung cancer. Alternatively, sequencing of non-small cell lung cancers in tobacco smokers vs. non-smokers identifies distinct genetic signatures. Tobacco induced tumors have a greater mutational burden than those found in non-smokers, which may in turn predict response to immunotherapy (Godwin et al., 2013; Hellmann et al., 2016).

### Alcohol

Alcohol can act in concert with tobacco to significantly increase the risk of cancer. Historically, alcohol was long associated with liver as well as head and neck cancers (LoConte et al., 2018). However, alcohol alone is now classified as a carcinogen, increasing the risk of several cancers in a linear dose dependent fashion, including breast, pancreas, liver, colon, esophagus and head and neck cancers. In hepatocellular cancers specifically, alcohol is known to induce chronic liver inflammation and fibrosis, which is itself a pre-cursor to malignancy (also discussed above). In addition, there is also evidence suggesting that patients with hepatocellular cancers and increased alcohol consumption show poorer outcomes once diagnosed with cancer (Barbara et al., 1992). More recently, alcohol has been associated with a host of other cancers. According to the American Society of Clinical Oncology (ASCO), between 5 and 6% of new cancers and cancer deaths globally can be “directly attributable to alcohol” (LoConte et al., 2018). Alcohol can cause direct DNA damage as a result of ethanol conversion to acetaldehyde as well as disrupt folate metabolic pathways (Seitz and Becker, 2007). With respect to the TME, evolving preclinical data suggests that ethanol can directly disrupt immune surveillance and innate immune response as well as induce reactive oxygen species (ROS) production and oxidative stress in CAFs (Sanchez-Alvarez et al., 2013). Alcohol may also disrupt the vascular endothelium thereby creating a microenvironment more permissive to metastasis and tumor migration (Xu et al., 2012). In breast cancer patients, particularly those with estrogen receptor (ER) positive breast cancers, Sanchez-Alvarez and colleagues suggest that ethanol induces ketone production in CAFs (Sanchez-Alvarez et al., 2013). In preclinical models, CAFs have also been shown to fuel tumor growth via oxidative mitochondrial metabolism and promote a more aggressive breast cancer phenotype (Donnarumma et al., 2017).

### Obesity

Historically, public health campaigns for cancer prevention have focused on tobacco's obvious link to cancer risk. More recently, however, ASCO suggests that obesity may soon outweigh tobacco as the leading modifiable risk factor for cancer (Ligibel et al., 2014). Not only does obesity increase the risk for a multitude of cancers but it may also decrease treatment delivery and worsen outcomes for those newly diagnosed. Frighteningly, over two thirds of the adult US population are overweight or obese and the number is growing (Flegal et al., 2002). Evolving data suggests that excess fat or “hyperadiposity” drives chronic inflammation, which in turn engenders a pro-tumorigenic milieu (Iyengar et al., 2016). White adipose tissue from obese patients is infiltrated by leukocytes, specifically macrophages, and T lymphoctyes (Underhill and Goodridge, 2012). When enlarged fat cells die, they release cytokines that recruit additional macrophages (Cinti et al., 2005). These macrophages then form a “crown” around the dying adipocytes, setting off an inflammatory cascade. The occurrence of these crown- like structures are commonly observed in obese patients with both breast and tongue cancers (Morris et al., 2011). Notably, Iyengar and colleagues have shown that even patients with a normal body mass index (BMI) but a relatively increased amount of body fat can also have inflammation in their breast tissue, indicating that BMI alone is not sufficient to predict for the influence of fat on body composition (Iyengar et al., 2017). Specifically, BMI is the ratio of a person's weight to height. This weight/height measurement alone does not reflect a more nuanced understanding of a person's body composition, or their relative percentages of fat and muscle. A seemingly lean person may be in fact “skinny fat,” wherein they have a relatively low BMI but their body composition is predominantly fat. Much of the work connecting obesity to cancer has been studied in the breast cancer population, because both fat and normal tissue in the breast can be readily evaluated as well as because of the relationship between fat and steroid/hormonal production. In breast cancer studies, inflamed fat tissue within the breast itself can increase local cytokine production, as well as expression of aromatase and ER gene expression (Cleary and Grossmann, 2009). Not surprisingly, obesity increases the risk of death among postmenopausal women with ER-positive breast cancer (Fuentes-Mattei et al., 2014). Obese mouse models suggest that activation of the AKT-mTOR pathway in the breast itself may specifically promote worse outcomes. Obesity has also been linked to poor outcomes for other types of cancers (Ligibel et al., 2014). In squamous cell cancer of the tongue for example, a diagnosis of obesity prior to tongue cancer diagnosis was associated with a five-fold increase of death (Iyengar et al., 2014).

### Dietary exposures and exercise

Given the relationship of obesity and cancer, diet and exercise modifications have gained increasing interest. Much of the work to date on the role of exercise, cancer, and inflammation has been done in the preclinical setting. Preclinical studies suggest that exercise can decrease inflammatory markers and modulate the TME (Koelwyn et al., 2015). Though the mechanisms by which exercise directly reduces inflammation are not entirely clear, possible hypotheses include decreasing IL-6, reduction in adipose tissue, and inhibition of TNFα (Koelwyn et al., 2015). Exercise may also increase the cytotoxicity and number of NK cells (Bigley and Simpson, 2015). Much like pharmacologic interventions, ongoing clinical trials are evaluating whether exercise as part of ongoing therapy may modulate tumorigenesis. Early data suggests that exercise leads to a reduction in visceral fat mass, decreased low grade chronic inflammation, and a subsequent reduction in pro-inflammatory adipokine secretion, as well as a reduction in macrophage infiltration into adipose tissue (Klionsky et al., 2016). In preclinical cancer models, exercise may decrease TAM and neutrophil infiltration and increase intratumoral cytotoxic T cell infiltration (Koelwyn et al., 2017). While the majority of the work in this space has been preclinical, a more recent randomized controlled trial evaluated the relationship of exercise and outcomes in breast cancer survivors. Specifically, this randomized controlled trial assessed the effects of a 16-week combined aerobic and resistance exercise intervention on metabolic syndrome, sarcopenic obesity, and serum biomarkers among sedentary, overweight, or obese survivors of breast cancer (Dieli-Conwright et al., 2018). Serum biomarkers included IGH1, insulin, IL-6, IL-8, TNFα, and steroid hormones (estrogen and testosterone). Sarcopenic obesity, BMI, and circulating biomarkers, including insulin, IGF-1, leptin, and adiponectin were significantly improved after exercise intervention (Dieli-Conwright et al., 2018). In addition to exercise, although data have not been entirely conclusive, some epidemiology studies have linked a higher fiber, lower fat diet to a decrease in some cancers. In breast cancer patients, for example, a high fiber, low fat diet results in lower circulating levels of estradiol among patients with a history of breast cancer, even in the absence of weight loss (Rock et al., 2004). Traditionally, designing and interpreting clinical trials to assess diet and cancer intervention have been challenging.

### Gut microbiota

More recently, gut bacteria have emerged as a possible link between metabolites in food and the TME. The immune system is dependent in part on exposure via the gut to microbiota as part of immunosurveillance (Kroemer and Zitvogel, 2018). Preclinical data suggests that diet can modify gut bacteria, specifically altering toll-like receptors on macrophages and dendritic cells as well as adipose inflammation (Garrett, 2015). Interestingly, in animal models, modification of gut bacteria affects CRC incidence and natural history (Song and Chan, 2017). Moreover, as reviewed by Song and Chan, a more “Western” diet, high in processed foods, red meat, processed sugars, and refined grains can lead to a dysregulated immune response and increased levels of inflammatory markers that is associated with a higher risk of colon cancer. Multiple, epidemiologic cross-cultural studies of diet and fecal bacteria indicate that diets high in fiber and low in fat alter gut bacteria and in turn are associated with a lower colon cancer risk. Alternatively, a diet high in vegetables, fruits, and whole grains lowers colon cancer risk. It is also well known that antibiotics can modify gut flora and recent data suggests that the use of repeated antibiotics may increase the incidence of lung, prostate, bladder and breast cancer possibly by altering gut flora (Velicer et al., 2004; Iida et al., 2013; Boursi et al., 2015). Evolving evidence suggests that gut bacteria may even influence response to immunotherapy. In melanoma patients, analysis of patient fecal microbiome samples indicated a higher diversity in bacteria and amount of Ruminococcaceae bacteria among those who responded to anti-PD-1 immunotherapy (Gopalakrishnan et al., 2018). Chaput and colleagues also demonstrated that gut microbiota, and in particular *Faecalibacterium* and other *Firmicutes* improved response to the CTLA-4 blockade by ipilimumab (Chaput et al., 2017). It is also hypothesized that some chemotherapies may uniquely alter gut bacteria and modulate immune response (Viaud et al., 2013).

### Infectious agents

In addition to modifiable behaviors associated with an inflammatory pro-tumorigenic cascade, several infectious agents have also been linked to cancer incidence (Moore and Chang, 2010). Globally, it is estimated that roughly 15% of all cancers are associated with infections (Parkin, 2006). Microbes (including bacteria and viruses) have been implicated in cancer in a number of different mechanisms, sometimes in combination. Possible mechanisms include direct DNA damage, via oncogenes or tumor suppressor inhibition, as well as via the promotion of chronic inflammation and in some instances, such as HIV, immunosuppression (Kuper et al., 2000). Common microbial associations with cancer include helicobacter pylori (gastric cancer and gastric MALT lymphoma), schistosoma haematobium (bladder cancer), HPV (human papillomavirus) (cervical and oral cancers), *clonorchis sinensis* (cholangiocarcinoma) and hepatitis B and C viruses (hepatobilliary cancers) to name a few (Kuper et al., 2000). However, the mechanisms by which each infection promotes cancer are not entirely linear. Hepatitis B and C viruses for example do not neatly fit into a category of direct or indirect carcinogens. Rather, it is likely that they contribute to carcinogenesis via a host of mechanisms including the introduction of viral products to the cancer cell as well as by inducing chronic inflammation (Tsai and Chung, 2010). As it is beyond the scope of this article, Moore and Chang provide an extensive review of the relationship between viruses and cancer (Moore and Chang, 2010). Similarly, the relationship between *H. Pylori* and gastric cancer is also multifactorial. *H. Pylori* both promotes inflammation of gastric epithelial cells and also induces specific protein changes and gene mutations (Chiba et al., 2008). Notably, not all patients with select infections go on to develop cancer. The ways in which infections promote cancer either by directly inducing changes to host cells or inducing a chronic inflammatory response varies significantly. In addition to efforts to eradicate infections with known cancer associations, such as with the HPV vaccine or *H. Pylori* treatment, it will be equally important to understand why some people are protected while others progress to infection-associated cancers.

### Intrinsic mechanisms leading to an inflammatory microenvironment

The mechanisms by which extrinsic factors can promote a pro-tumorigenic environment are inextricably linked to the ways in which genetic and epigenetic alterations can aide a cancer cell escape host defense mechanisms. Importantly, oncogenic mechanisms require a tight bidirectional cross talk of cancer cells with their microenvironment mediated by the production of chemokines, cytokines, growth factors, prostaglandins, ROS and nitrogen oxygen species (NOS), as well as recruitment of inflammatory cells into the tumor tissue. Many of the powerful oncogenes possess the ability to initiate a signaling cascade resulting in an inflammatory response in the proximity of the cells that harbor those oncogenes. The discovery that many oncogenic drivers are deeply involved in the modulation of a pro-oncogenic microenvironment and inflammatory processes suggested possible paracrine effects where altered expression or activity of the same genes in a stromal and/or immune cell may dictate epithelial fate and vice versa.

### Tumor-elicited inflammation, oncogenes and tumor suppressors

The advent of high-throughput sequencing techniques has led to the identification of hundreds of genetic and epigenetic alterations in genes associated with signaling pathways involved in cancer. Historically, genetic and functional studies have focused on a better understanding of the consequences of oncogenic activation in the context of the tumor cell itself and thus far only a limited number of studies has assessed their contribution beyond the cell-intrinsic effects. Here, we review common oncogenic and tumor suppressor pathways that contribute to tumor-associated inflammation (Figure 1). These include receptor and non-receptor tyrosine kinases (TKs), RAS signaling, TP53, APC, and PTEN.

### Tyrosine kinases

The activity of (receptor) TKs is central to many cellular processes. TKs also play cardinal roles in cytokine function, and are crucial for the signal transduction of various pro- and anti-inflammatory cytokines such as TNFα, IL-6, and IL-10. Due to their central status, TKs have received heightened attention as therapeutic targets, partially due to their potential to combat the chronic inflammatory state associated with many malignancies such as rheumatoid arthritis (RA), cardiovascular diseases, and cancer. In solid and blood cancers, TKs are frequently mutated leading to ligand-independent constitutive activation of downstream signaling pathways. For example, more than 90% of the non-leukemic classical myeloproliferative neoplasms (MPNs) are clearly driven by abnormal JAK2 activation, especially the cytokine receptor/JAK2 pathways and their downstream effectors (Passamonti et al., 2011). In line with a crucial role of JAK2 in cytokine signal transduction, MPN patients are characterized by high levels of pro-inflammatory cytokines in their circulation, which can be reduced by JAK inhibitor therapy (Verstovsek et al., 2010; Geyer et al., 2015; Mondet et al., 2015). Indeed, it is believed that the impressive clinical activity of ruxolitinib is a result of its anti-inflammatory effects. This suggests that aberrant JAK-STAT pathway activation is important for the induction and maintenance of the inflammatory state in MPN patients. Notably, whether *JAK2* is mutated or not, the efficacy of ruxolitinib is comparable (Deininger et al., 2015). We have recently shown that both mutant and non-mutant hematopoietic cells are the source of pro-inflammatory cytokines in MPN mouse models and patients and that JAK-STAT signaling in mutant and non-mutant cells has to be inhibited in order to achieve therapeutic response (Kleppe et al., 2015b). This data suggests that sequential, interlinked, and selective steps, which bear clear resemblance to tumor-cell-organ microenvironment interactions commonly found in solid cancer and metastasis, also drive aberrant cytokine production in hematological malignancies. Other TKs which may be involved in the induction of an inflammatory state include c-Kit, EGFR, PDGFR, RET, VEGFR, c-Fms, and FGF (extensively reviewed in Yang and Karin, 2014).

### RAS signaling

The RAS superfamily of small GTPases comprises a group of more than 150 small G proteins. RAS proteins are signal transduction molecules central to many cellular processes. Mutations in one of the three canonical *RAS* genes, H-*RAS*, N-*RAS*, and K-*RAS*, are among the most common genetic abnormalities in human cancers. It is well established that aberrant RAS activation drives neoplastic transformation by influencing diverse aspects of the malignant phenotype in a cell autonomous manner, most importantly cell proliferation, survival, and mobility. Interestingly, more recent reports suggest that the role of oncogenic RAS extends beyond the effects on the tumor cell itself. Oncogenic Ras causes genotoxic stress and senescence in cells (Coppé et al., 2008). Intriguingly, senescent cells are known to secrete a myriad of inflammatory factors. In line, oncogenic Ras has been shown to accelerate and amplify a senescence-associated secretory phenotype that largely depends on IL-8 and IL-6 secretion thereby promoting tumorigenesis through effects on non-transformed cells during the process of inducing senescence (Coppé et al., 2008). Interestingly, high Ras activity in pancreatic acinar cells leads to cellular senescence and is sufficient to induce an inflammatory phenotype that is similar to the histological features of chronic pancreatitis suggesting that mutant K-*ras* is a cause rather than a secondary effect of chronic pancreatitis (Ji et al., 2009). Notably, patients with chronic pancreatitis have an increased risk of developing pancreatic cancer and K-*RAS* mutations are commonly found in chronic pancreatitis (Lüttges et al., 2000), but also observed in hyperplastic ducts within normal pancreas (Tada et al., 1996). Indeed, a large proportion of the adult human population possesses *RAS* mutations in tissues besides the pancreas, including colon and lung. Ras-mediated cytokine production has been repeatedly linked to activation of the inflammatory regulator NF-κB. It has been shown that in presence of mutant Ras, inflammatory stimuli initiate a NF-κB-dependent positive feedback loop involving Cox-2 resulting in prolonged Ras signaling and chronic inflammation and precancerous lesions in mice (Daniluk et al., 2012). In keratinocytes, expression of oncogenic Ras instigates an autocrine loop through IL-1α, IL-1R, and MyD88 leading to phosphorylation of IκBα and NF-κB activation (Cataisson et al., 2012). Moreover, activation of oncogenic Ras has been shown to enhance expression of squamous cell carcinoma antigens 1 and 2 and IL-6 via the NF-κB pathway (Catanzaro et al., 2014). K-*RAS* mutations mediate therapeutic resistance and are associated with poor prognosis, and until now, no effective anti-RAS inhibitor has reached the clinic (Cox et al., 2014). Given the growing body of evidence linking aberrant RAS and NF-κB it is intriguing to speculate that the NF-κB pathway could be exploited as potential preventive and therapeutic target in cancers harboring mutant *RAS*.

### TP53

TP53 is a stress-responsive transcription factor (TF) and acts as a major tumor suppressor inhibiting neoplastic transformation by preventing the escalation of chronic tissue imbalance (Cooks et al., 2014). TP53 is a central hub for diverse stress signals, including ROS and NOS, cytokines, and infectious reagents (Cooks et al., 2014). TP53 also participates in the control of multiple cell cycle checkpoints. Mutations disabling TP53 tumor suppressor functions are the most frequent events in human cancer. For example, molecular alterations of *TP53* are a defining feature of ovarian high-grade serous carcinomas (Cancer Genome Atlas Research, 2011; Vang et al., 2016). In addition to the cell autonomous effects of TP53 inactivation/dysfunction, compelling evidence suggests that *TP53* missense mutants may not merely lose their tumor suppressive functions, but can also acquire new oncogenic properties through the activation of cell non-autonomous pathways. Specifically, multiple studies have linked mutant TP53 (such as *TP53* p.R273H) and chronic inflammation to tumorigenic progression through different molecular interactions, including NF-κB (Cooks et al., 2013; Di Minin et al., 2014; Cui and Guo, 2016). In line, it has been demonstrated that *TP53* mutants interact directly with NF-κB and that both factors impact the other's binding at diverse sets of active enhancers thus promoting a unique enhancer landscape of cancer cells in response to chronic inflammation (Rahnamoun et al., 2017). Moreover, clinical studies in primary breast carcinoma, head and neck squamous cell carcinoma, and CRC suggest that *TP53* inactivation or deletion induces inflammation (Yin et al., 1993; Brentnall et al., 1994; Hussain et al., 2000; Linderholm et al., 2000; Lee et al., 2007). The effects of Tp53 loss have also been studied in diverse mouse models. For example, in a mouse model of prostate cancer, Tp53 loss resulted in enhanced transcription of cytokines and chemokines, accumulation of ROS and protein oxidation products, enhanced macrophage activation and neutrophil clearance, hypersensitivity to LPS, and high expression of metabolic markers (Komarova et al., 2005). Further, Lujambio and colleagues showed that Tp53-deficient hepatic stellate cells secrete factors that stimulate polarization of macrophages into a tumor-promoting M2 state leading to increased liver fibrosis and accelerated transformation of adjacent hepatocytes (Lujambio et al., 2013). Collectively, inhibition of tumor-associated inflammation is likely another important tumor suppressive function of TP53.

### APC

CRC represents a paradigm for the link between inflammation and cancer (Lasry et al., 2016). Patients with inflammatory bowel disease, such as ulcerative colitis, are more likely to develop CRC and non-steroidal anti-inflammatory drugs show strong preventive effects (Chia et al., 2012). In mouse models of colitis, genetic, and functional studies have shown that inflammation alone suffices for tumor development and that inflammation-induced DNA damage can link chronic colitis and tumor initiation. Moreover, colorectal tumors exhibit tumor-elicited inflammation and upregulation of inflammatory signature genes (Wang and Karin, 2015). In fact, the type, density, and location of immune cells within human colorectal tumors have proven to be a reliable measure of patient outcome (Galon et al., 2006; Grivennikov et al., 2010; Norton et al., 2015). Inactivating mutations in *APC*, resulting in aberrant β-catenin activation, are found in 80% of all human colon cancers. In addition, *APC* loss predisposes humans to familial adenomatous polyposis, an autosomal dominant syndrome, in which patients develop numerous colorectal polyps (Groden et al., 1991). The tumor suppressor activity of APC has been extensively studied in the setting of epithelial transformation. In mice, the presence of an autosomal dominant *Apc* mutation in intestinal epithelial cells (IECs) leads to tumor development upon inactivation of the other allele due to spontaneous loss of heterozygosity (LOH) (Moser et al., 1990, 1993; Jackstadt and Sansom, 2016). Colon-specific deletion of *Apc* leads to formation of colorectal tumors with upregulation of pro-inflammatory cytokines. Interestingly, this work suggests that epithelial barrier defects and microbial invasion into the TME leads to an activation of IL-23 producing myeloid cells, which, in turn, drive IL-17 mediated tumor growth (Grivennikov et al., 2012). Chronic NF-κB activation in IECs has been shown to lead to the development of intestinal adenomas linking inflammation and tumorigenesis (Greten and Karin, 2004; Vlantis et al., 2011). In line with a pro-tumorigenic function of NF-κB in CRC, crossing transgenic mice with chronic epithelial NF-κB activation to Apc^Min/+^ mice leads to accelerated LOH and intestinal tumor initiation through iNOS up-regulation (Shaked et al., 2012). Intriguingly, little is known about the consequences of APC inactivation in immune cells. While it has been shown that *Apc* mutant mice are characterized by an altered intestinal immune homeostasis and impaired control of inflammation by regulatory T lymphocytes (Gounaris et al., 2009; Akeus et al., 2014; Chae and Bothwell, 2015), only a recent study demonstrated that Apc inactivation in T-cells renders the immune system unable to tackle gut inflammation due to deficient T-cell activation (Agüera-González et al., 2017).

### PTEN

The phosphatase PTEN (phosphatase and tensin homolog deleted on chromosome 10) functions as tumor suppressor by inhibiting PI3K-dependent cellular proliferation, survival, growth, and motility. PTEN function is frequently disrupted in human cancer, but has also been shown to play a role in other diseases (Leslie and Downes, 2004). The phosphatase has originally been identified as tumor suppressor through its mutation leading to abolished or greatly decreased phosphatase activity. More recently, it has been shown that PTEN protein expression is lost in a greater number of patients as originally expected based on mutational frequency. Both genetic and epigenetic mechanisms are discussed as possible mechanisms causing loss of PTEN protein expression in the absence of coding sequence mutations (Leslie and Downes, 2004). PTEN represents another tumor suppressor that has been shown to exhibit its oncogenic functions, at least in part, through manipulation of the microenvironment by triggering the release of inflammatory mediators from tumor cells. For example, PTEN dysfunction has been shown to increase the expression and signaling of pro-inflammatory chemokine CXCL8 in prostate cancer cells that resulted in a coordinated response of both tumor and stromal cells. Increased release of Cxcl8 from *Pten*-deleted tumor cells augmented the sensitivity and responsiveness of tumor cells to stromal chemokines by concurrently inducing the upregulating of chemokine receptors on tumor cells and inducing stromal chemokine production (Maxwell et al., 2014). In one study, progression of *Kras* mutant PDAC was associated with deletion and loss of expression of *Pten*. Interestingly, Pten loss and activation of K-Ras cooperated and accelerated pancreatic cancer development by promoting NF-κB activation and its cytokine network, which in turn promoted stromal activation and immune cell infiltration (Ying et al., 2011). Similarly, Kim and colleagues showed that knockdown of tumor suppressor *Pten* and *Tp53* in breast cancer cells synergized to activate a pro-inflammatory Il-6/Stat3/NF-κB signaling axis (Kim et al., 2015). In addition, loss of *Pten* has been shown to prevent anti-tumor immunity (Spranger et al., 2015; Peng et al., 2016). In conclusion, available data suggests that loss of *PTEN* leads to activation of an inflammatory loop that contributes to malignant transformation.

### Genetic studies of the tumor microenvironment

About two decades ago, Fattaneh Tavassoli and his team were the first to report genetic alterations, specifically LOH at microsatellite markers, in the stroma of mammary carcinomas (Moinfar et al., 2000). Since then a number of human studies have analyzed the mutational spectrum of selected tumor suppressors and LOH/allelic imbalances of specific markers. In 2001, Kurose and colleagues analyzed invasive LCM-procured epithelium and stroma from adenocarcinoma samples of the breast and reported that both epithelial and stromal cells harbor LOH of specific markers including those at 10q23 (in the vicinity of *PTEN*), 17p13–p15 (in the vicinity of *TP53*) and 16q24 with a higher frequency in the neoplastic epithelial compartment (Kurose et al., 2001). Around the same time, a different group reported LOH on chromosome 17p13, 3p25-26, and 9q32-33 in the stroma of invasive urothelial carcinoma (Paterson et al., 2003). In 2002, Kurose et al. reported that mutations in the tumor suppressors *PTEN* and *TP53* occur at a high frequency in the neoplastic breast epithelium and/or stroma (Kurose et al., 2002). Charis Eng and her team followed then up on their work with a larger study analyzing the mutational status of *TP53* and LOH in 218 invasive breast cancers patients (Patocs et al., 2007). They found a high frequency of *TP53* mutations in hereditary (49%) and sporadic tumor (27.4%) stroma. Similarly, 60% (hereditary) and 51% (sporadic) of the patients carried LOH or allelic imbalances in the stroma. In addition to previous studies, the authors related their genetic findings to clinical and pathological features of the disease. Interestingly, in the sporadic group, the presence of *TP53* mutations in the stroma was associated with lymph node status and nodal metastasis. Those observations suggest that genetic alteration of *TP53* in the stroma may accelerate tumor growth. Overall, numerous independent investigators have described a variety of genetic, epigenetic, and genomic alterations in the stroma of a broad variety of solid tumors and inflammatory conditions (Man et al., 2001; Tuhkanen et al., 2004; Hu et al., 2006; Ishiguro et al., 2006; Kim et al., 2006; Bian et al., 2007; Joshua et al., 2007; Weber et al., 2007; Yagishita et al., 2008). While such findings still remain controversial (Allinen et al., 2004), it challenges the current paradigm that the microenvironment, albeit aberrant, would not be targeted by genetic alterations and highlights the necessity to further our mutational understanding of this crucial component. Importantly, the finding of genetic and epigenetic changes in the stroma raises a number of questions, regarding the mechanisms leading to these genetic lesions, the populations in which they are found, their functional importance to tumor development, and clinical implications for patients with mutations in the stroma. Using a variety of different approaches, multiple groups have tried to model how modulation of known tumor suppressors and/or oncogenes in stromal cell types may affect malignant transformation using mouse models. Early work by the group of Terry van Dyke suggested that oncogenic stress mediated by an initial driver event in the epithelium would create pressure in the microenvironment that leads to loss and selection of a Tp53-deficient stromal compartment (Hill et al., 2005). This is in line with the knowledge that stromal fibroblasts with intact Tp53 can render the microenvironment less supportive of the survival and spread of adjacent tumor cells by secretion of a spectrum of growth inhibitors (Komarova et al., 1998; Moskovits et al., 2006). Intriguingly, Hill and colleagues further showed that loss of *Tp53* in the stromal compartment disrupts the homeostasis between the epithelial and stromal tissues ultimately leading to loss of *Tp53* also in the tumor suggesting that stromal loss may actually precede epithelial *Tp53* loss (Hill et al., 2005; Palumbo et al., 2015). About a decade later, Farmaki and colleagues also showed that the TME induces strong selective pressure onto stromal cells, selecting specific subpopulations of stromal fibroblasts that can survive and expand more efficiently within the TME (Farmaki et al., 2012). Notably, higher numbers of cancer cells were associated with a stronger proliferative advantage of *Tp53*-deficient fibroblasts as compared to wild-type cells in line with the concept that loss of *Tp53* heightens the sensitivity of mutant fibroblasts to epithelial-derived growth factors. Mechanistic studies revealed that the oncogenic effect of *Tp53*-deficient microenvironment is mediated by enhancing the levels of inflammatory cytokines/chemokines and immunosuppressive molecules, which disturbed immune cell composition and exacerbated immunosuppressive function within the microenvironment (Guo et al., 2013). Further, ablation of Tp53 in fibroblasts has been shown to promote tumor growth in a murine prostate cancer model (Addadi et al., 2010). While TP53 has been the major focus of most mechanistic studies, it is conceivable that mutational inactivation of other tumor suppressors as well as epigenetic alterations such as histone modifications and DNA methylation may be responsible for the generation of stromal cells with pro-tumorigenic properties (Hu et al., 2005; Peng et al., 2005; Bar et al., 2009). It will be important to uncover the identity and regulation of secreted factors that are responsible for the tumor cell-induced inhibition of stromal TP53 induction and other potential tumor suppressors.

### Clonal hematopoiesis and inflammation

Leukocytes represent a crucial component of the inflammatory TME. The various leukocyte subsets, including macrophages, neutrophils, basophils, and lymphocytes can interact with each other, but also with non-hematopoietic stromal cell types and epithelial tumor cells, thereby orchestrating tumor progression and invasiveness. To date, many groups have studied the prognostic value of infiltrating immune cells in solid tumors (reviewed by Barnes and Amir, 2017; Hammerl et al., 2017). With the increasing interest in harnessing the immune system to treat cancer with checkpoint inhibitors and other novel agents, a better understanding of the composition of the immune infiltrate as prognostic marker is of increasing importance. However, the simple presence of a specific immune cell type in the TME does not predict their function. For example, macrophages cover a continuum of functional states that allows them to fulfill different tasks depending on the microbial and cytokine milieu. Further complicating the scenario, recent data challenge the paradigm that the integrity of the genome of immune cells is intact in solid cancer patients. At first, a large body of genetic data emerged demonstrating that elderly individuals without signs of overt leukemia harbor somatic mutations in hematopoietic cells leading to expansion of mutant blood cells. Most of the mutations were identified in genes encoding for known leukemia drivers such as the chromatin modifiers *TET2, ASXL1*, and *DNMT3A* (Busque et al., 2012; Genovese et al., 2014; Jaiswal et al., 2014; Shlush et al., 2014; McKerrell et al., 2015; Young et al., 2016; Buscarlet et al., 2017). Not surprisingly, follow up studies showed that patients with clonal hematopoiesis (CH) are at an increased risk of developing hematological malignancies. However, the same study showed that patients with CH are also at an increased risk of atherosclerotic cardiovascular disease compared to individuals without CH (Jaiswal et al., 2014, 2017a,b). Atherosclerotic cardiovascular disease has long been thought of as an inflammatory disease; however, only recently, data from the CANTOS study was published reporting that selectively targeting inflammation by using a therapeutic monoclonal anti-IL-1β antibody can reduce cardiovascular risk (Ridker et al., 2017a). But is there a mechanistic link between the presence of mutant cells in the blood, the development of atherosclerotic cardiovascular diseases, and inflammation? Intriguingly, a growing body of evidence suggests a causal link between mutations in epigenetic modifiers seen in CH and inflammation. For example, *Tet2*-deficient macrophages exhibit an increase in inflammasome-mediated IL-1β secretion, which is associated with accelerated development of atherosclerosis in these mice (Fuster et al., 2017). *Tet2* also seems to exhibit a suppressive role in the regulation of immunity and inflammation, independent of its role in DNA methylation and hydroxymethylation, but by repression of transcription via histone deacetylation (Zhang et al., 2015). Specifically, loss of Tet2-mediated gene transcription resulted in increased expression of inflammatory mediators upon injection of the mice with the highly potent inflammatory stimulus LPS. Further, *Tet2*-deficient mice were more susceptible to experimental colitis and endotoxin shock (Zhang et al., 2015). *Dnmt3a*-deficient mast cells display an increased sensitivity to acute and chronic inflammatory stimulation (Leoni et al., 2017). Yet in another immune cell type, both Dnmt3a and Tet2 seem to be important for the regulation of macrophage activation, polarization and inflammation (Yang et al., 2014; Li et al., 2016). It is likely that this is just the tip of the iceberg and the coming years will provide a greater understanding of the functional and regulatory roles of these important epigenetic regulators in different immune compartments, myeloid malignancies, solid tumors, and non-malignant inflammatory diseases.

The accumulating body of evidence that older individuals have clinically unapparent CH, together with the increasing awareness of the importance of microenvironment in tumor progression, we hypothesized that immune cells infiltrating tumors might be characterized by clonally selected mutations. Targeted sequencing analysis of leukocytes isolated from a small number of tumors from treatment naïve breast cancer patients demonstrated that indeed infiltrating CD45-positive hematopoietic cells harbor somatic mutations in cancer genes, including *BCOR, TET2, DNMT3A*, in a subset of patients (Kleppe et al., 2015a). These mutations were not found in peripheral blood cells, admixed tumor cells, or epithelial germline samples. This finding suggests that mutant infiltrating leukocytes may interact with cancer cells, which has significant clinical implications for tumor development and response to treatment. Our data was partially corroborated by a recent report that hematopoietic cells are also genetically abnormal in a fraction of patients with advanced solid cancers even at a younger age (Coombs et al., 2017). Coombs and colleagues analyzed paired tumor and blood samples from ~8,000 patients with advanced solid cancers. In total, one fourth of all patients carried at least one CH mutation in the blood sample, with 4.5% of the patients harboring presumptive driver mutations (CH-PD). CH was associated with increasing age, tobacco use, and prior radiation therapy. Further, patients with CH had an increased risk of hematologic cancers and CH-PD was associated with a shorter survival. Notably, the primary cause of death was progression of the primary non-hematopoietic tumor. While both studies support the intriguing idea that immune cells are genetically abnormal which could be of utmost importance from a diagnostic and therapeutic standpoint, there are fundamental differences in the study design and the conclusions. Regardless, neither study data discerns between the different leukocyte subsets. As such, it will be critical to assess the distribution and frequency of CH mutations within the different hematopoietic/immune compartments.

## Prevention and therapeutic intervention

### Checkpoint blockade immunotherapy

Historically, there has been a longstanding clinical interest in the overlap between the immune system and cancer. Most famously, in the early nineteenth century, William Coley developed a “Coley's Vaccine,” a concoction of bacteria, after noticing tumor regression among patients who developed high fevers from *Steptococcus pyogenes* infection. His treatments and the research therein largely fell out of favor with the advent of surgical advances, radiation, and chemotherapy. In the last few years, however, the growing understanding of the tumor microenvironment and interplay between the immune system and cancer has dramatically changed the landscape of immunotherapy options. In addition to investigational efforts into vaccines and oncolytic viruses, several immunotherapy treatments have recently been approved. Specifically, an understanding of immune response and activation in cancer, and in particular the role of immune blockade by CTLA-4 (cytotoxic T lymphocyte-associated protein 4) and PD-1/PD-L1 (programmed cell death protein 1/programmed cell death protein ligand 1) has revolutionized the treatment of several types of cancers, including but not limited to melanoma, non-small cell lung cancer, urothelial, head and neck, and renal cancers. Ipilimumab was the first anti- CTLA-4 antibody to be approved in advanced melanoma (Sharma and Allison, 2015). In determining which cancers benefit from checkpoint inhibition, evidence suggests that those with a higher mutational burden (such as in response to tobacco) and in some instances microsatellite instability-high (MSI-h) tumors may respond best (Pleasance et al., 2010; Snyder et al., 2014; Le et al., 2015). Not surprisingly, given the activation of the immune system with these therapies, oncologists have had to become increasingly facile with managing a host of immune related side effects (Postow and Hellmann, 2018; Postow et al., 2018). For a more extensive review of clinically relevant checkpoint inhibitors and immunotherapy please see: (Farkona et al., 2016).

### Non-steroidal anti-inflammatory drugs (NSAIDs)

COX enzymes (COX1 and COX2) are the primary targets of non-selective non-steroidal anti-inflammatory drugs (NSAIDs), which include aspirin, indomethacin, piroxicam, sulindac, and ibuprofen. Inhibition of COX enzymes results in the inhibition of prostaglandins, which play important roles in many physiological processes. NSAIDs are commonly used for the treatment of fever, pain, and swelling. Slowly, albeit still somewhat controversial and largely based on epidemiologic studies, NSAIDs have emerged as drugs with potential anti-cancer activity which may decrease the incidence and mortality of colon, breast, stomach, and lung cancers (reviewed in Ulrich et al., 2006, Figure 2). For example, chronic use of aspirin has been suggested to reduce the risk of pro-inflammatory conditions such as inflammatory bowel disease and the risk to develop colorectal cancer (Chia et al., 2012). Similarly, different studies suggest that ibuprofen might also stop certain cancers from developing (Harris et al., 2003; Johnson et al., 2010). Overall, a large body of observational data regarding a protective effect of NSAIDs from developing certain cancers, specifically CRC, is strong. A recently performed meta-analysis investigated the relationship between NSAIDs and lymph nodes/distant metastasis. The study suggests that NSAIDs hold potential in the management of cancer metastasis, regardless of whether NSAIDs are used at pre-diagnosis or post-diagnosis (Zhao et al., 2017). At this point, it cannot be denied that anti-inflammatory compounds may represent novel, less toxic, agents for cancer therapy, nonetheless, carefully designed, controlled, blinded, and randomized trials are required to create a benefit-risk assessment and address many outstanding questions such as the lowest effective dose, the age at which to initiate therapy, the duration of treatment, and which population benefits of NSAIDs chemopreventive activity. Toward this end, the AspECT trial, a large phase III, randomized study is designed to assess the long-term chemoprevention effect of esomeprazole in combination with or without aspirin in patients with Barrett's metaplasia (NCT02070757).

**Figure 2:**
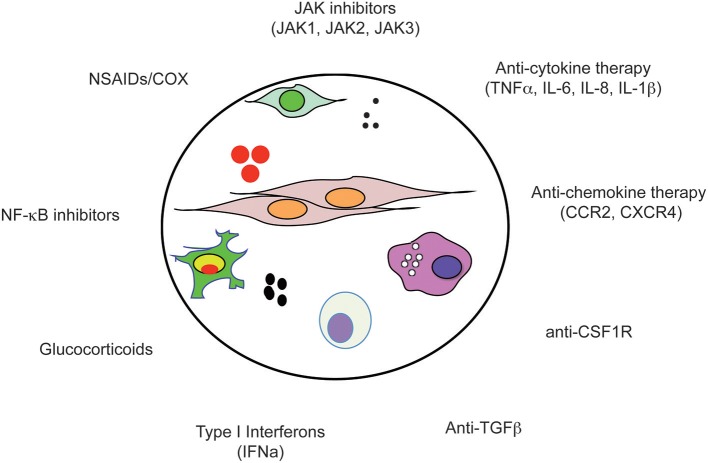
Anti-inflammatory drugs. The tumor microenvironment (circle) is composed of tumor cells, fibroblasts, endothelial cells, pericytes, and various cells associated with the immune system such as macrophages and NK cells. Importantly, within the microenvironment a tight bidirectional cross talk of cancer cells with their microenvironment occurs which is mediated by the production of chemokines, cytokines, growth factors, prostaglandins, ROS, and NOS, as well as recruitment of inflammatory cells into the tumor tissue. Due to the complex nature of the microenvironment, a large number of druggable dependencies have been identified and are currently under investigation as therapeutic targets for anti-inflammatory drugs.

### Glucocorticoids

Glucocorticoids (GCs) have been used in the clinic for over half a century. Indeed, due to their strong anti-inflammatory and immunosuppressive properties, GCs are the most prescribed immune suppression medications worldwide. Orally inhaled GCs are commonly used to suppress various allergic, inflammatory, and autoimmune disorders. For example, they are by far the most effective drugs for the treatment of asthma, which is largely due to their efficacy to inhibit inflammatory cytokine gene expression (Barnes, 2011). GCs exhibit multiple modes of action and interfere with the function of basically all immune cell types. For example, GCs suppress cytokine release and cell migration, induce apoptosis, and change cell differentiation fates (Perretti and Ahluwalia, 2000). In oncology, GCs have only shown modest efficacy in breast and prostate cancer and not in other cancer types. In general complexity and controversial observations are associated with GC treatment in non-hematologic cancer types (Lin and Wang, 2016). Nonetheless, GR antagonists are currently tested in several clinic trials in combinations with chemotherapy, including breast, prostate, and lung cancer. In hematological cancers, GCs such as dexamethasone have proven astonishingly effective in the treatment of lymphoid neoplasms including acute lymphoblastic leukemia, chronic lymphocytic leukemia, MM, Hodgkin's lymphoma, and non-Hodgkin's lymphoma where GCs induce growth arrest and apoptosis. GC-induced apoptosis of lymphoid cells is sought to be induced via multiple signaling pathways including Bim, a member of the Bcl2 family, and suppression of cytokines via inhibition of the activity of different TFs such as NF-κB (Lin and Wang, 2016). In contrast, only little is known about the mode of action of GCs in solid cancers.

### JAK inhibitors

The Janus family of kinases (JAKs) comprises four members: JAK1, JAK2, JAK3, and TYK2. JAKs are critical for the signal transduction of about 60 different cytokines that rely on type I and II cytokine receptors. Many of these cytokines are central to the growth of malignant cells and drivers of immune-mediated diseases. Consequently, JAKs have emerged as a new class of pharmacologic agents. The first selective JAK inhibitor to enter clinical trials was tofacitinib. Tofacitinib potently inhibits JAK3 and JAK1 and to a lesser extent JAK2 and shows a high degree of kinome selectivity (O'Shea et al., 2013). Tofacitinib is the first JAK inhibitor approved for the treatment of moderate to severe RA and active psoriatic arthritis. Mechanistically, as a JAK inhibitor, tofacitinib efficiently blocks the biological effects of common γchain cytokines including IL-2, IL-4, IL-15, and IL-21 and consequently suppresses allergic Th2 responses (Fukuyama et al., 2015). For example, in a Th2-dependent asthma mouse model, tofacitinib reduces pulmonary eosinophilia (Kudlacz et al., 2008). Further, tofacitinib has been shown suppress the differentiation of pathogenic Th1 and Th17 cells as well as innate immune signaling by limiting the production of pro-inflammatory cytokines in a LPS-induced sepsis model (Ghoreschi et al., 2011). Interestingly, tofacitinib and ruxolitinib, a JAK1/2 inhibitor, are currently being tested in patients with skin and hair disorders, including the autoimmune disease alopecia areata, and mild to moderate atopic dermatitis (Bissonnette et al., 2016; Mackay-Wiggan et al., 2016; Liu et al., 2017). Also other pharmacologic agents targeting different JAK family members have found increasing attention as anti-inflammatory targets in different disease contexts. Ruxolitinib (Jakafi) was the first FDA approved JAK inhibitor for the treatment of myelofibrosis (MF) and, more recently, for patients with polycythemia vera (PV) who have had an inadequate response to hydroxurea (Verstovsek et al., 2010; Raedler, 2015). Ongoing clinical trials also assess the efficacy of ruxolitinib in patients with post-MPN AML and CML with minimal residual disease, another form of MPN (Eghtedar et al., 2012; Pemmaraju et al., 2015; Assi et al., 2018). Interestingly, on a mechanistic level, recent work from Tarafdar and colleagues suggests that CML stem cells downregulate MHC-II, allowing them to evade the host immune response. They found that this deregulation can be reverted by JAK inhibition and IFNγ (Tarafdar et al., 2017). Besides ruxolitinib, other JAK2 inhibitors are also under clinical development for the treatment of MPNs (Kontzias et al., 2012) and their strong anti-inflammatory potential provides a rationale for repurposing these drugs as solid tumor therapeutics (Quintás-Cardama et al., 2011; Plimack et al., 2013; Buchert et al., 2016). However, clinical studies of different JAK inhibitors in solid tumors have been marked by lack of activity. For example, a phase 1 study of JAK1/2 inhibitor AZD-1480 in solid tumor patients was discontinued due to unusual adverse side effects and lack of clinical activity (Plimack et al., 2013). A comprehensive table listing clinical trials conducted with JAK inhibitors can be found in Buchert et al. (2016). Despite initial excitement, in 2016 Incyte discontinued several clinical trials due to insufficient level of efficacy including: the phase III study (JANUS 2) of ruxolitinib or placebo plus capecitabine in patients with advanced or metastatic pancreatic cancer, the phase II sub-study of ruxolitinib in patients with metastatic colorectal cancer and low CRP, and the phase II studies in breast and lung cancer. In addition, Incyte discontinued the investigation of INCB39110, a selective JAK1 inhibitor as first-line treatment for metastatic pancreatic cancer, however, preclinical studies still continue. The JAK/STAT pathway also presents new potential targets in graft-vs.-host disease (GvHD). Despite major improvements in allogeneic hematopoietic stem cell transplantation (HSCT), GvHD still remains a matter of concern, especially if patients show no adequate response to systemic corticosteroid. Corticosteroid-refractory (SR) acute and chronic GvHD is associated with poor prognosis and therapeutic options for salvage therapy are needed. Due to its strong anti-inflammatory properties, multiple groups have performed retrospective studies to assess the potential of ruxolitinib as salvage therapy in steroid resistant GVHD patients (Zeiser et al., 2015; Mori et al., 2016; Khandelwal et al., 2017). The data suggests that while low-dose ruxolitinib shows potential in this setting, it will be important to determine in prospective studies the optimal dose to achieve the best-tolerated dose and an optimized tapering schedule to avoid withdrawal symptoms which are also observed in MF patients upon discontinuation of the drug. Overall, JAK inhibitors have a wide range of indications due to their central role in the regulation of cytokine signaling. It can be expected that the market for JAK inhibitors will continue to grow in the coming years and, similarly, the field will probably move toward using novel strategies to achieve more specific and versatile inhibition of this important family of kinases.

### Anti-cytokine therapy

Cytokine and cytokine receptors have been recognized as excellent drug targets for a variety of diseases characterized by chronic inflammation due to their important function as rate-limiting signaling molecules (Feldmann, 2008). TNFα blockade in patients with rheumatoid arthritic was the first major success demonstrating the beneficial effect of anti-cytokine therapy in a disease associated with chronic inflammation (Elliott et al., 1993; Feldmann and Maini, 2001). The role of TNFα and IL-6 as master regulators of tumor-associated inflammation and tumor-promoting functions makes them promising targets for adjuvant anti-cancer therapy (Yan et al., 2006; Grivennikov and Karin, 2011). Clinically, elevated TNFα serum levels have been detected in patients with a wide range of tumor types, although TNFα is not universally detectable. Notably, high levels of TNFα have been correlated with tumor stage, extent of para-neoplastic complications, and worse survival. Due to a prospective study showing that TNFα and IL-6 levels correlate with the degree of disease, and PSA progression in prostate cancer patients, TNFα and IL-6 are currently suggested as additional markers that reflect the activity level of this disease (Michalaki et al., 2004). Three biologics targeting TNFα are currently approved including etanercept, infliximab, and adalimumab and different small molecules that inhibit TNFα signaling or synthesis (for example thalidomide) are under development. TNFα blocking agents may not only find a place as anti-tumor agents, but may have a role in controlling the severe cancer pain associated with metastatic bone lesions. Similarly, anti-IL-6 therapy holds potential to alleviate cancer-related symptoms. Several early phase clinical studies with IL-6 targeting agents currently support the hypothesis that IL-6 may be indeed an effective anti-cancer target. Several humanized monoclonal antibodies against soluble and membrane bound IL-6R (tocilizumab, REGN88) or against IL-6 (siltuximab and sirukumab) are currently in clinical trials at various development stages for several types of cancer including MM, metastatic renal cell carcinoma, B-cell lymphoproliferative diseases (Heo et al., 2016). A fully human anti-IL-8 antibody, ABX-IL8, has shown promising therapeutic efficacy in preclinical models (Yang et al., 1999; Huang et al., 2002) however the therapeutic value of blocking IL-8 has yet to be assessed in cancer patients. Excitingly, recently published data from the CANTOS study, a randomized trial of the role of IL-1β inhibition in atherosclerosis, suggests that anti-inflammatory therapy targeting the IL-1β pathway could significantly reduce incidence of lung cancer and lung cancer mortality (Ridker et al., 2017b). CANTOS was not formally designed as a cancer detection or treatment trial and will need to be replicated; however, there is precedent for other cancer types (MAPp1, NCT01767857; Lust et al., 2009; Hong et al., 2014; Goel et al., 2016). In contrast to the success stories of anti-TNFα and anti-IL-6 targeting studies, little clinical success has been observed with therapeutics targeting single cytokines in other diseases such as COPD or asthma. One can speculate that the lack of clinical success of therapeutic targeting single cytokines or cytokine receptors in other inflammatory diseases may be due to the redundancy of the cytokine network and thus required to target more than one cytokine at a time. This may also be true for the treatment of different types of cancer; however, it is too early to say what place anti-cytokine therapy will claim in the field of oncology.

### Interferons

IFNs present another group of cytokines which exhibit anti-tumor activity and can activate the immune system and are leveraged in the clinic (reviewed in Parker et al., 2016). Type I IFNs, IFNα and IFNβ, can be produced and released by tumor cells and by most cell types in the human body (Parker et al., 2016). In contrast, IFNγ, a type II IFN, is primarily expressed by T cells and NK cells. Regardless of their source, all IFNs can act via intrinsic and extrinsic mechanisms thereby exerting direct antitumor effects or indirect effects through antitumor immune responses. Tumor cell intrinsic effects of IFNs are a result of their capability to regulate the expression of genes involved in various cellular processes such as cell growth, proliferation, and survival. For example in patients with PV, IFNα has been shown to inhibit the growth of JAK2V617F-mutant hematopoietic stem and progenitor cells via activation of the p38 MAPK pathway (Lu et al., 2010). Tumor cell extrinsic effects of IFNs are mediated through their ability to influence the activity of nearly every immune cell type including T cells, NK cells, monocytes, and macrophages (Hervas-Stubbs et al., 2011). Recent work by Parker and colleagues showed that intact type I IFN signaling is required to induce anti-metastatic immunity by NK and CD8 T cells. Interestingly, their work further showed that the tumor cells themselves were a source of type I IFNs and that suppression of the tumor cells own IFN production functions as immune-evasion mechanism (Bidwell et al., 2012). IFNs exert their immunoregulatory effects on the cells of the immune system through the regulation of tumor antigens on tumor cells (Greiner et al., 1984) and antigen presentation by MHC complexes and ligands for receptors of immune checkpoints (Propper et al., 2003; Schreiner et al., 2004). Further, IFNs can regulate immunity by triggering the release of “downstream” chemokines, cytokines, and interleukins such as IL-15 (Nguyen et al., 2002; Burkett et al., 2004). Besides their role in immunity, IFNs also promote angiogenesis and osteoclastogenesis which are important to tumor growth (Cheon et al., 2014). Unlike most cytokines which are targeted with antagonists, IFNα is used as therapeutic protein in the treatment of patients with some types of solid cancer, including Kaposi sarcoma, melanoma, and renal cell carcinoma, and different hematologic malignancies such as MPNs, and viral diseases (Kirkwood, 2002). The first type of IFN-based therapy showed striking results on the survival rates of patients with hairy cell leukemia and CML (Platanias, 2013; Stein and Tiu, 2013). While the discovery of the Philadelphia chromosome as genetic alteration in CML and the arrival of BCR-ABL targeting TKIs in the clinic have largely replaced the need for IFNα-based treatment in this disease, some recent clinical studies have shown that combination of imatinib and IFNα is superior to either therapy alone, perhaps due to the fact that IFNα targets preferentially CML stem cells (Talpaz et al., 2013). Notably, due to its success in numerous clinical trials, IFNα is used as first-line treatment choice for patients with high-risk PV (Barbui et al., 2011; Falchi et al., 2015). Regardless of the specific use, IFN-based therapies have their limitations due to dose-limiting side effects. As a direct consequence, various other strategies such as the use of IFNα conjugated with polyethylene glycol moieties or pattern recognition receptor agonists to stimulate type I IFN production in patients are under development aimed at increasing efficacy over toxicity (Parker et al., 2016).

### CSF1R inhibition

In contrast to IFN-based therapy, colony-stimulating factor 1 receptor (CSF1R) has only recently emerged as a cancer drug target. CSF1 is essential for the development and maintenance of macrophages, and as such provides a critical target for depleting these cells within the TME. The efficacy of CSF1R inhibition, and its capacity to deplete TAMs seems to vary depending upon tumor type and tissue. Administration of BLZ945, a small molecule inhibitor of CSF1R, in a murine model of glioma led to regression and long term increases in survival (Pyonteck et al., 2013). Surprisingly, this reduction in tumor burden was not associated with TAM depletion, but rather TAMs underwent “re-education,” an alteration in gene expression profile with decreased expression of M2-associated anti-inflammatory markers. Meanwhile breast cancer models utilizing either BLZ945 or CSF1R neutralizing antibodies led to depletion of TAMs with limited therapeutic efficacy as a monotherapy (DeNardo et al., 2011; Shiao et al., 2015; Olson et al., 2017). Understanding why an environment is permissive to maintaining TAMs in the face of CSF1R inhibition will be critical for clinical implementation. One possibility is the expression of GM-CSF, IFNγ, and CXCL10, factors that were shown to rescue macrophage from BLZ945-mediated killing (Pyonteck et al., 2013). Whether these factors are also causatively involved in “re-education” remains to be determined. While CSF1R inhibition as a monotherapy has shown various model dependent results, combination therapy with cytotoxic agents has shown broader applicability. In murine breast tumor models, combinations of CSF1R inhibitors such as PLX3397 or BLZ945 with radiotherapy or paclitaxel displayed greater efficacy than with either single agent alone (DeNardo et al., 2011; Shiao et al., 2015; Olson et al., 2017). This combined efficacy does not appear to be limited to cytotoxic therapy as in a murine model of pancreatic adenocarcinoma PLX3397 treatment synergized with immune checkpoint blockade (Zhu et al., 2014). In this setting, treatment with PLX3397 led to a reduction in macrophages, where the remaining cells underwent a reprogramming not dissimilar from that seen in gliomas with BLZ945 treatment (Pyonteck et al., 2013). Similar combined efficacy was also seen in glioma models combining CSF1R inhibition with multiple TKIs (Yan et al., 2017). In another study, prolonged CSF1R inhibition led resistance through macrophage-derived IGF1 signaling and PI3K activation in tumor cells (Quail et al., 2016). In this setting, combination CSF1R and PI3K/IGF1R inhibition was capable of preventing relapse. Collectively, these studies demonstrate that CSF1R inhibition shows promise in chemo sensitizing combination therapies and in combination with TKIs. Multiple clinical trials are currently ongoing evaluating CSF1R inhibition with checkpoint immunotherapy, radiation therapy, and targeted small molecules (Cannarile et al., 2017). In addition to identifying the optimal tumor types for CSF1R inhibition, proper dosing schedules will also need to be determined. While the goal of many Phase I trials is to identify the maximum tolerable dose of the drug, care should be taken to understand the different outcomes of high dose and low dose CSF1R inhibition. It is possible, that at high doses, CSF1R inhibitors may deplete macrophages even in “protective” environments, and that lower doses may offer a capacity to “re-educate” macrophages without depleting them in the TME. Proper biomarkers, potentially derived from gene expression studies following CSF1R inhibition, will be necessary to determine the optimal dose based on the desired outcome of depletion vs. reprogramming.

### Inflammation and drug resistance

Diverse resistance mechanisms to targeted cancer therapy have emerged and present one of the foremost challenges in cancer today. Drug resistance may pre-exist (intrinsic, primary drug resistance) or may be acquired under the strong selective pressure during the course of treatment (acquired resistance) (Zahreddine and Borden, 2013). Importantly, some of these resistance pathways lead to multi-drug resistance (MDR), generating an even more difficult clinical problem to overcome. Alterations in the level of cytokines, chemokines, and growth factors have emerged as yet another mechanism conferring resistance to chemotherapeutic treatments (Jones et al., 2016). For example, mounting evidence suggests a crosstalk between IL-6 and MDR in cancer and potential therapeutic opportunities arising from this role of IL-6 (Ghandadi and Sahebkar, 2016). Since the 1990s, elevated serum levels of IL-6 have been associated with worse survival in breast cancer patients (Zhang and Adachi, 1999; Salgado et al., 2003; Knüpfer and Preiss, 2007). Subsequently, functional studies revealed that autocrine production of IL-6 by tumor cells confers resistance to several chemotherapeutic agents, suggesting that acquisition of the ability of tumor cells to produce IL-6 represents another self-protective mechanism (Conze et al., 2001). Further, increased secretion of IL-6 has been linked to resistance to bortezomib in MM and to etoposide and cisplatin in hormone-independent prostate carcinomas (Borsellino et al., 1995; Frassanito et al., 2001; Voorhees et al., 2007). IL-6-induced STAT3 feedback activation in response to the EGFR inhibitor erlotinib has been associated with the development of resistance and poor prognosis in lung adenocarcinoma (Lee et al., 2014). Work published by Korkaya and colleagues suggests that trastuzumab resistance may be mediated by an IL-6 inflammatory loop (Korkaya et al., 2012). Importantly, soluble factors mediating drug resistance such as IL-6 can be released by the tumor cell itself or the TME. For example, Ara and colleagues demonstrated that in neuroblastoma the bone marrow microenvironment is a source of IL-6 and sIL-6R, thereby allowing cancer cells to escape the cytotoxic effects of multiple chemotherapeutics (Ara et al., 2013). Regardless of the source of the cytokine IL-6, IL-6 targeting strategies promise to be effective in combination with cytotoxic agents, TKIs, or targeted antibody therapy to prevent drug resistance. Besides IL-6, IL-8 has emerged as another resistance cytokine. Similarly to IL-6, clinical studies have linked high serum levels of IL-8 to disease progression of various cancer types (Xie, 2001). In line, by using cytokine antibody arrays to identify cytokines associated with drug resistance, two independent groups concordantly found that IL-6 and IL-8 are key markers for the development of drug resistance (He et al., 2011; Shi et al., 2012). The list of “resistance cytokines” is long including AMF (autocrine mobility factor), AM (adrenomedullin), IL-4, and IL-10 (Jones et al., 2016). In addition, a recent study showed that patients with increased levels of the inflammatory biomarkers ferritin and C-reactive protein (CRP) had a markedly poorer response to trastuzumab-containing therapy (Alkhateeb et al., 2012). Another important factor to consider when discussing the relation between cytokines and cancer drug resistance is the fact that the inflammatory master regulator NF-κB is activated by a variety of cytotoxic chemotherapy agents including cisplatin, paclitaxel, docetaxel, and doxorubicin (Nakanishi and Toi, 2005; Li and Sethi, 2010). Anticancer therapeutic induced NF-κB activation often leads to the activation of pro-survival signaling pathways. In addition, NF-κB is often constitutively active in response to a variety of cancer-promoting agents. Both the constitutive and therapy-induced NF-κB activation eventuates in drug-resistant tumors, at least in part, by the induction of inflammatory cytokine secretion. Consequently, NF-κB signaling has emerged as an attractive molecular target for pharmacological intervention and its inhibitors as potential sensitizer to anticancer drugs; however, despite the clinical success in newly diagnosed and relapsed/refractory multiple myeloma (MM) and mantle cell lymphoma patients, the NF-κB inhibitor bortezomib (Valcade) has fallen short of original expectations (Lin et al., 2010; Godwin et al., 2013). Paradoxically, in some instances bortezomib has been shown to activate NF-κB signaling and stimulate cytokine secretion. For example, exposure to bortezomib of prostate cells led increased expression and release of IL-8. Mechanistically, bortezomib increased the accumulation of IκB kinase β (IKKβ) in the nucleus and increased recruitment of nuclear IKKβ, phosphorylated p65, and transcription factor early growth response-1 (EGR1) to the IL-8 promoter (Singha et al., 2014). Given that IL-8 is another resistance-associated cytokine this may explain why bortezomib is less effective in certain tumor types. Overall, there is a strong link between inflammation and drug resistance and beyond their ability to prevent or decrease chemotoxicity, anti-inflammatory agents may also have therapeutic effects when combined with conventional agents, acting additively or synergistically, or sensitizing cancer cells to treatment with conventional cancer therapies. In order to optimize the therapeutic window and regimens of anti-inflammatory cancer therapy, clinical oncologists, and experimental cancer researchers will need to identify for each drug the appropriate molecular target, cancer type, disease stage, and treatment duration. Based on the current state of knowledge, it can be expected that anti-inflammatory agents will be most effective in combination with anti-angiogenic, cytotoxic, and cytostatic agents.

### Future direction

Herein we discuss the constituents of the TME, the causes of inflammation within, and therapeutic strategies aimed at disrupting the signaling pathways critical to bolstering cancer development. Given the evolutionary nature of cancer, it is unlikely that any single therapeutic option will lead to durable cures or eradication of complex tumors. Indeed combinations therapies targeting tumor cell intrinsic oncogenic pathways, and TME-derived support pathways will offer the best means to effectively eradicate disease. These combinations are not trivial, as the TME itself is an evolving system with relatively understudied kinetics in the clinical setting. Effective therapeutic intervention may require new paradigms of dosing and schedule to identify therapeutically optimal schedules that may be non-linear and independent of maximum tolerable doses. These arduous studies may benefit from preclinical animal model development, but will inevitably require clinical testing. To expedite studies, the development of biomarkers sensitive to TME-targeted is of the utmost importance. Without these tools, potentially viable compounds and strategies may be left behind due to suboptimal dosing or rather suboptimal understanding of the correct dosing paradigm. While the molecules and pathways described here may provide sound mechanistic and molecular targets for clinical development, unveiling their full potential will require an integrated understanding of the systems-level effects of inflammation. Instead of strict molecular pathways, inflammatory microenvironments can be thought of as ecological landscapes and causes of inflammation as selective pressures sculpting tumor evolution along these landscapes. This ecological landscape is both dynamic and systemic, extending beyond the simple genetics within malignant tumor cells and beyond invasive tumor edge.

## Author contributions

MK has written most of the part of this review dealing with prevention and therapeutic invention as well as intrinsic mechanisms. RB has written most of the part on cellular constituents of an inflammatory microenvironment. EC has written most of the part of this review dealing with external factors and exposures causing inflammation. All authors have corrected the whole manuscript.

### Conflict of interest statement

The authors declare that the research was conducted in the absence of any commercial or financial relationships that could be construed as a potential conflict of interest.

## Abbreviations

AML: acute myeloid leukemia
APC: adenomatous polyposis coli
ASCO: American Society of Clinical Oncology
BMI: body mass index
CAFs: cancer-associated fibroblasts
CANTOS: Canakinumab Anti-inflammatory Thrombosis Outcome Study
CH: clonal hematopoiesis
CH-PD: CH with a presumptive driver mutation
COPD: chronic obstructive airway disease
ER: estrogen receptor
FDA: Food and Drug Administration
GCs: glucocorticoids
GI: gastrointestinal
GvHD: graft-versus-host disease
NOS: nitrogen species
HPV: human papillomavirus
HSCT: allogeneic hematopoietic stem cell transplantation
IECs: intestinal epithelial cells
IFN: interferons
JAKs: janus family of kinases
LOH: loss of heterozygosity
LPS: lipopolysaccharides
MDSCs: macrophage-derives suppressor cells
MDR: multi-drug resistance
MM: multiple myeloma
MPNs: myeloproliferative neoplasms
NSAIDs: non-selective non-steroidal anti-inflammatory drugs
PDAC: pancreatic ductal adenocarcinoma
Pten: phosphatase and tensin homolog
PV: polycythemia vera
RA: rheumatoid arthritis
ROS: reactive oxygen species
TAMs: tumor associated macrophages
TF: transcription factor
TKs: tyrosine kinases
TKIs: tyrosine kinase inhibitors.
